# Review of traditional uses, botany, chemistry, pharmacology, pharmacokinetics, and toxicology of Radix Cyathulae

**DOI:** 10.1186/s13020-019-0237-x

**Published:** 2019-04-30

**Authors:** Yongliang Huang, Shanshan Wang, Li Liu, Wei Peng, Jiaolong Wang, Ying Song, Qianghua Yuan, Xing Yuan, Chunjie Wu

**Affiliations:** 1 Teaching Hospital of Chengdu University of Traditional Chinese Medicine, Chengdu, 610072 China; 2 Chengdu University of Traditional Chinese Medicine, Chengdu, 610075 China

**Keywords:** Cyathulae Radix, Traditional usages, Origin, Phytochemistry, Pharmacology, Pharmacokinetics, Toxicology

## Abstract

Cyathulae Radix (CR), also known as “Chuanniux” is a well-known traditional Chinese herbal medicine that has been used in China for thousands of years. The present work reviewed advances in traditional uses, origin, chemical constituents, pharmacology, pharmacokinetics, and toxicity studies of CR. This work aims to provide more up-to-date references for modern study and application of this plant. Furthermore, the possible trends and perspectives for future research of this plant are also discussed. In China, the roots of CR have been widely used in clinical practice to treat orthopedic, gynecological, and urologic diseases. Currently, over 59 compounds have been isolated and identified from CR, including alkaloids and flavonoids. The extracts and compounds from CR have many pharmacological activities both in vivo and in vitro. They provide beneficial effects on the hematological system and anti-inflammatory properties. However, few studies have investigated the pharmacokinetics and toxicity of CR. Further studies should be undertaken to investigate the clinical effects, toxic constituents, and pharmacokinetics of CR; perform quality evaluation; and establish quality criteria for processed *C. officinalis*. Furthermore, studying the changes of raw and processed CR and the variety of this plant between different cultivated areas and cultivars will be interesting.

## Introduction

Cyathulae Radix is a perennial herbaceous plant distributed in Sichuan, Guizhou, and Yunnan provinces in China. Its root is commonly used as a traditional Chinese herbal medicine called “Chuanniux.” It is traditionally believed to have the effects of removing “blood stasis,” antifertility, anti-inflammation, stimulating menstruation, and curing orthopedic diseases including bone injury [1].

Cyathulae Radix has been listed in the Pharmacopoeia of the People’s Republic of China. Since 1953, over 100 prescriptions containing CR have been utilized to treat gynecological diseases, orthopedic diseases, blood stasis diseases, stroke, and deficiency of the liver and kidney [2–4]. CR has various pharmacological activities, including anti-inflammatory effects, effects on the hematological system and immune system, and antihypertensive effects. Many phytochemical studies have been performed on RC, and over 59 compounds have been identified and isolated from this herb, including alkaloids, flavonoids, tannins, triterpenes, and fatty acids. Currently, CR remains a traditional Chinese medicine (TCM) listed in the Chinese Pharmacopoeia, and cyasterone is used as the indicator agent to characterize its quality [1].

In the present paper, the advances in traditional utilization, origin, phytochemistry, pharmacology, and toxicity of CR are systematically reviewed. Relevant literature on CR was collected from Chinese medicine books; articles from Ph.D. and MSc. Dissertations; and scientific databases, including PubMed, ScienceDirect, Web of Science, Springer, Elsevier, Wiley, and CNKI. We systematically reviewed the multi-faceted literature on CR, including the origin and advances of phytochemistry, pharmacology, and pharmacokinetics. Furthermore, possible research and application directions and new perspectives on CR are also discussed.

## Traditional usages

Given its wide spectrum of pharmacological activities, CR has long been used as a medicinal plant in China. In TCM, CR has been used as a wind-damp-dispelling and blood-stasis-removing medicine [5] (Anonymous, 1963). The root of CR is the main part used as a medicine named Chuan Niu Xi. In addition to raw root, CR is commonly processed by stir-baking with yellow rice wine to produce a yellowish or dark brown color. The raw root and wine fried root are the most common clinically used forms [6, 7] (Anonymous, 1977, 2015). The medicinal use of this plant, first listed in Xianshou Lishang Xuduan Mifang in the Tang dynasty in China, dates back to 1170 years ago [8]. In Shennong Bencaojing Shu, the root of CR was described as a herbal medicine with good diuretic and nourishing properties. In the Qing dynasty, another famous TCM monograph Depei Bencao described the root of CR as a herbal medicine with the effects of nourishing the liver and kidney and activating blood to remove stasis. It was described as a treatment for rheumatism, chronic malaria, diarrhea, drench pain, hematuria, stethalgia, and abdominal pain. CR was also described as a medicinal plant used for tonifying the liver and kidney, strengthening the bones and muscles, promoting diuresis, relieving stranguria, and removing blood stasis in other monographs of materia medica in China, including Benjing Fengyuan, Bencao Qiuzhen, and Bencao Zhengyi [3].

Currently, the root of CR is commonly used as an important TCM for the treatment of various orthopedic diseases (e.g., bone injury, osteoarthritis, rheumatic arthritis, and arthralgia), gynecological disease, and urologic diseases in decoction and proprietary Chinese medicine [3, 6]. In the 2015 edition of the Pharmacopoeia of the People’s Republic of China, the root of CR was recorded as a traditional Chinese medicinal material and contained in over 30 prescriptions [9].

## Origin

Cyathulae Radix is a herbaceous perennial plant. It reaches approximately 50–100 cm in height and contains many branches. The stems of this plant are erect and four prismatic but circular at the bottom. They exhibit green color, which is sometimes mixed with purple. The stems are covered with sparse coarse hairs. The leaves are elliptic to narrowly elliptic, opposite, and petiolate. The leaf margins are entire. The leaves are 3–13 cm long and 1.5–5 cm wide and covered with thick lodging coarse hairs on the pressure side and long pubescence hairs on the reverse side. The flowers have a green–white color and are clustered in inflorescences. The compound cymes gather into flower pellets with a diameter 1–1.5 cm. The flower pellets interactive opposite on rachis. The bisexual flowers are located in the center, whereas sterile flowers are located on both sides. The bracts are ovoid and approximately 4–5 mm in length. The bisexual flowers are 3–5 mm in length with lanceolate sepals. The flower has five stamens and five staminodes. The base of the stamen is densely villous. The staminode is rectangular in shape, 0.3–0.4 mm long, and 0.1–0.2 mm wide. The pistil is ovary superior and 1-loculed and has a fine style. The utricles are long elliptical, obovate, and 2–5 mm long. The seeds are ovoid, lentoid, and 1.5–2 mm long. CR flowers from June to July and sets fruit from August to September. The roots of CR are nearly cylindrical, slightly twisted, and 30–60 cm long and have a diameter of 0.5–3 cm. The underpart of the root is slightly slender and has few branches. The surface root is yellowish brown or grayish brown with longitudinal wrinkles, branch marks, and many horizontal long leather-like protuberances. The roots are pliable but strong; they have a light yellow or brown section. The vascular bundles are point-like and arranged in several rounds of concentric rings. The stele is large in the transverse section. The outer tough vascular bundles are intermittently arranged in 4–11 rounds, and the inner stratification layer is visible in the inner vascular bundles. The xylem catheter normally has a single radial arrangement and is ligneous. Wood fiber is developed, and parenchyma cells contain calcium oxalate crystal [1]. Different from other plants of the same genus, the cylinder number of tertiary vascular bundles in 1-year-old CR roots can reach up to six laps, and that in 2-year-old CR roots can reach up to eight laps [10].

This plant is native to South and Southwest China. At present, it is mainly cultivated in Sichuan and Chongqing. The plant is also distributed in some provinces, including Guizhou, Hubei, Hunan, Shanxi, Gansu, Jiangxi, and Zhejiang. The altitude in the cultivation areas ranges from 1150 to 2680 m [11, 12]. The plant has two close relatives, *C. capitata* (Wall.) Moq. and the hybrid of *C. officinalis* and *C. capitata* (Wall.) Moq., which are common in origin and widely found in markets. Their roots are identified as adulterant of Chuanniuxi [13]. The main differences between CR and its adulterants are the shape of inflorescence, roots, and taste of the root. Compared with CR, *Cyathula capitata* (Wall.) Moq. has larger and longer inflorescence and exhibits a taupe color after being dried. The roots are thick, large, and always lignified and have an acrid taste. The hybrid of these two species also has a large inflorescence with a black gray color after being dried. The roots taste bitter and cause numb tongue, and they have multiple branches at the bottom. In addition, the duramen of the root is hard [14]. The leaf morphology and microscopic features of these three plants are different [15]. Genetic diversity analysis shows large genetic differences between CR and its adulterants. However, molecular marker technology can easily distinguish them. In 2010, CR and its adulterants were identified by SCAR markers. The primer SC-495 is effective for distinguishing CR and *C. capitata* (Wall.) Moq. [16].

## Phytochemistry

**Fig. 1:**
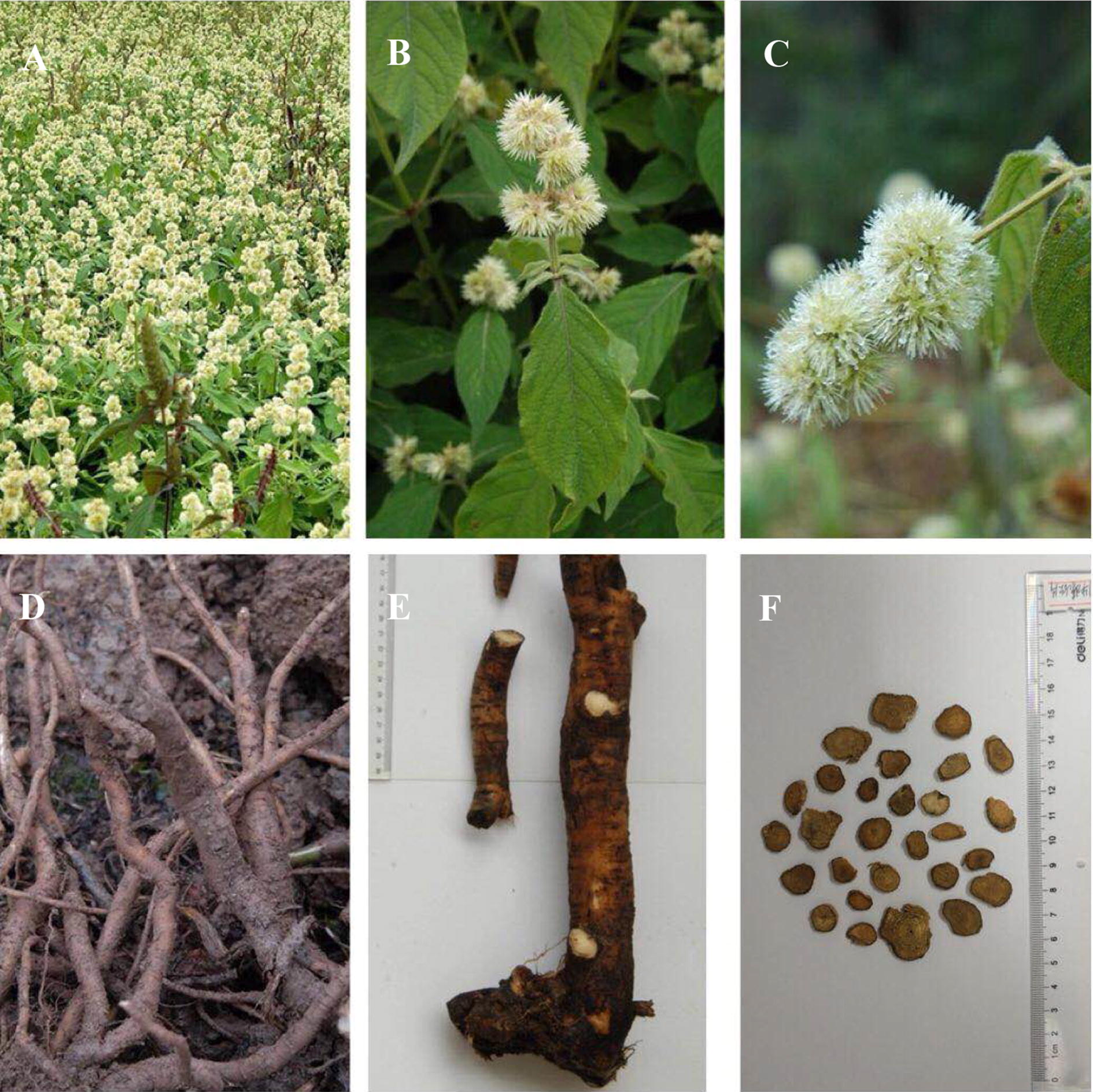
Cyathulae Radix (**A**). Flower of Cyathulae Radix (**B**, **C**). Roots of Cyathulae Radix (**D**, **E**). Transverse section of Cyathulae Radix (**F**)

Many chemical compounds have been isolated from CR. The chemical components in CR can be roughly divided into three categories: triterpenoid saponins, steroid ketones, and polysaccharide and other compounds (Fig. 1).

### Triterpenoid saponins

Triterpenoid saponins are composed of three saponins and sugar molecules. Tetracyclic triterpenoids and pentacyclic triterpene are common. Sugar that is connected to the glycosides usually contains glucose, rhamnose, glucuronic acid, and arabia sugar. Saponins found in CR are pentacyclic triterpene saponins. Three terpenoid saponins, including oleanolic acid type three terpenoid saponins, ivy saponins, and bamboo saponins, have been reported in CR (Table 1) [17].

**Table 1:** Saponins reported from CR

No.	Compound name	Reference
1	3-*O*-β-d-glucopyranosyl-(1 → 2)-α-l-rhamnopyranosyl-(1 → 3)-β-d-glucopyranosyl-28-*O*-β-d-glucopyranosyl oleanolic acid	Zhou et al. [18, 19]
2	3-*O*-β-d-glucopyranosyl-(1 → 2)-[α-l-rhamnopyranosyl-(1 → 3)]-β-d-glucopyranosyl-28-*O*-β-d-glucopyranosyl oleanolic acid	Zhou et al. [18, 19]
3	3-*O*-α-l-rhamnopyranosyl-(1 → 3)-β-d-glucuronopyranosyl-28-*O*-β-d-glucopyranosyl oleanolic acid	Zhou et al. [18, 19]
4	3-*O*-β-d-glucuronopyranosyl-28-*O*-β-d-glucopyranosyl oleanolic acid	Zhou et al. [18, 19]
5	3-*O*-β-d-glucopyranosyl-(1 → 2)-α-l-rhamnopyranosyl-(1 → 3)-β-d-glucuronopyranosyl oleanolic acid	Zhou et al. [18, 19]
6	3-*O*-α-l-rhamnopyranosyl-(1 → 3)-β-d-glucuronopyranosyl oleanolic acid	Zhou et al. [18, 19]
7	3-*O*-β-d-glucuronopyranosyl oleanolic acid	Zhou et al. [18, 19]
8	3-*O*-α-l-rhamnopyranosyl-(1 → 2)-[β-d-glucopyranosyl(1 → 3)]-β-d-glucuronopyranosyl-28-*O*-β-d-glucopyranosyl oleanolic acid	Zhou et al. [18, 19]
9	3-*O*-α-l-rhamnopyranosyl-(1 → 4)[β-d-glucopyranosyl-(1 → 2)]-β-d-glucuronopyranosyl oleanolic acid or 3-*O*-α-l-rhamnopyranosyl (1 → 3)[β-d-glucopyranosyl-(1 → 2)]-β-d-glucuronopyranosyl oleanolic acid	Zhou et al. [18, 19]
10	3-*O*-α-l-xylopyranosyl-(1 → 3)-β-*D*-glucuronopyranosyl-28-*O*-β-d-rhamnopyranosyl oleanolic acid	Montoya et al. [20]
11	3-*O*-[α-l-rhamnopyranosyl-(1-2)-β-d-glucuronopyranosyl]-28-*O*-[β-d-glucopyranosyl-(1-6)-β-d-glucopyranosyl] hederagenin	Montoya et al. [20]
12	3-*O*-β-d-glucopyranosyl-α-l-rhamnopyranosyl-β-d-glucuronopyranosy1-28-*O*-α-l-xylopyranosyl hederagenin	Montoya et al. [20]
13	3-*O*-α-l-rhamnopyranosyl-(1-4)-β-d-glucuronopyranosyl-28-*O*-β-d-glucopyranosyl hederagenin or 3-*O*-α-l-rhamnopyranosyl-(1 → 3)-β-d-glucuronopyranosyl-28-*O*-β-d-glucopyranosyl hederagenin	Montoya et al. [20]
14	3-*O*-β-d-glucuronopyranosyl-28-*O*-β-d-glucopyranosyl hederagenin	Montoya et al. [20]
15	3-*O*-α-d-rhamnopyranosyl-(1→3)-β-d-galactopyranosyl-(1 → 3)-β-d-glucuronopyranosyl hederagenin	Montoya et al. [20]
16	3-*O*-β-d-glucuronopyranosyl-28-*O*-α-l-rhamnopyranosyl hederagenin	Montoya et al. [20]
17	3-*O*-β-d-glucopyranosyl-α-l-rhamnopyranosyl-β-d-glucuronopyranosyl-28-*O*-β-d-glucopyranosyl gypsogmin	Montoya et al. [20]
18	3-*O*-β-d-glucopyranosyl-(1 → 2)-[α-l-rhamnopyranosyl-(1 → 3)]-β- d-glucuronopyranosyl gypsogmin	Montoya et al. [20]
19	3-*O*-α-l-rhamnopyranosyl-β-d-glucuronopyranosyl 28-*O*-β-d-glucopyranosyl gypsogmin	Montoya et al. [20]
20	3-*O*-[α-l-rhamnopyranosyl-(1 → 3)-(*n*-butyl-β-d-glucopyranosiduronate)]-28-*O*-β-d-glucopyranosyl oleanolic acid	Zhou et al. [18, 19]
21	3-*O*-[α-l-rhamnopyranosyl-(1 → 3)-(β-d-glucuronopyranosyl)] oleanolic acid	Zhou et al. [18, 19]
22	3-*O*-[β-d-glucopyranosyl-(1 → 2)-α-l-rhamnopyranosyl-(1 → 3)-β-d-glucuronopyranosyl]-28-*O*-β-d-glucopyranosyl oleanolic acid	Zhou et al. [18, 19]
23	3-*O*-β-d-glucopyranosyl oleanolic acid	Zhou et al. [18, 19]
24	28-*O*-β-d-glucuronopyranosyl-(1 → 4)-β-d-glucopyranosyl hederagenin	Zhou et al. [18, 19]

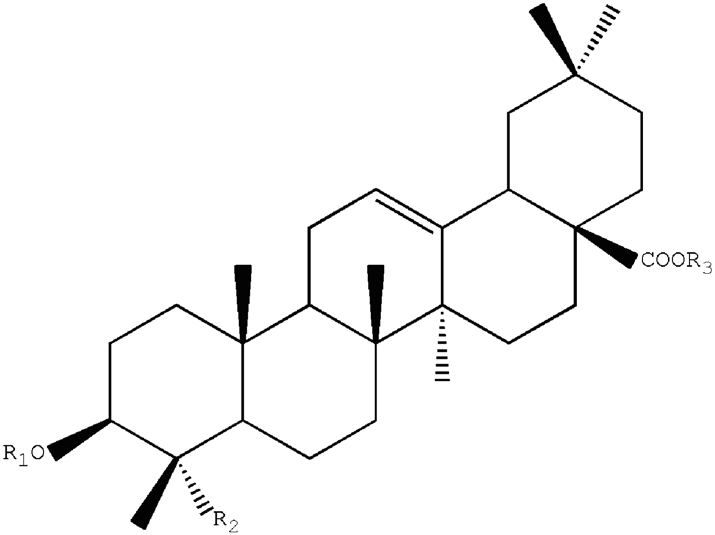
 
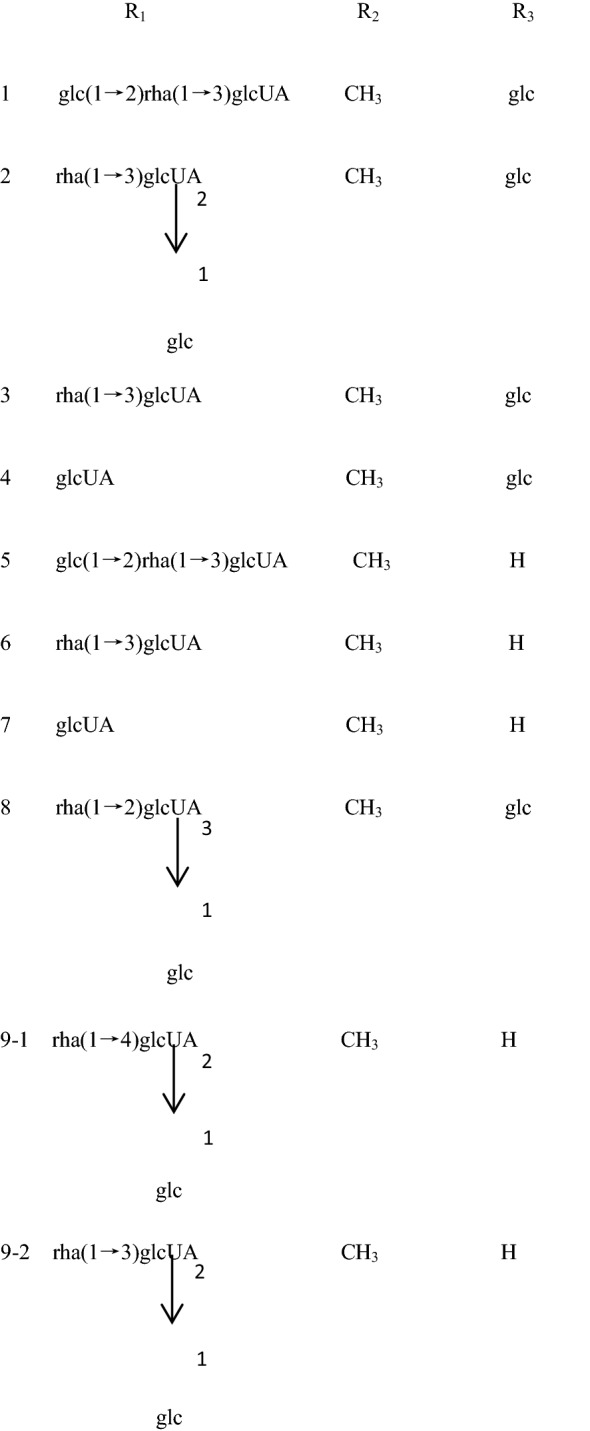
 
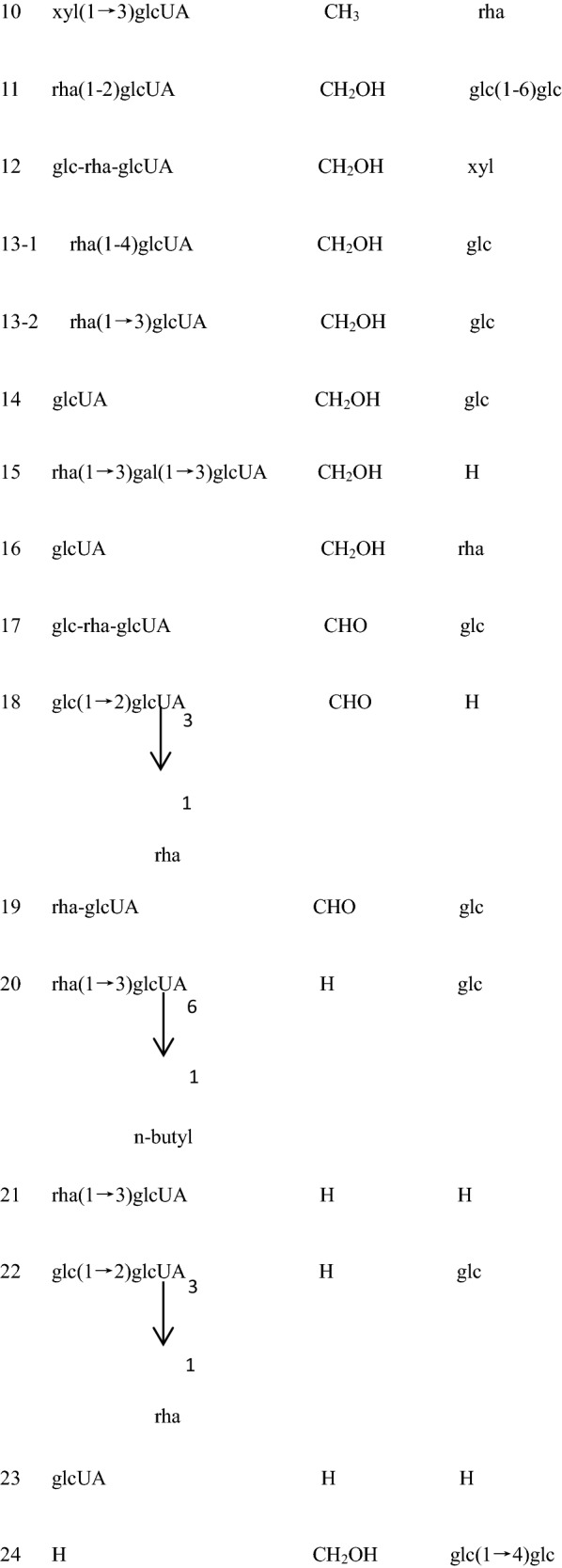

### Steroid ketones

Steroid compounds are another important kind of active compounds in CR (Table 2).

**Table 2:** Phytosterones discovered from CR

No.	Compound name	Reference
1	Cyasterone	Zhou et al. [18, 19]
2	Isocysterone	Zhou et al. [18, 19]
3	28-Epi-cyasterone	Zhou et al. [18, 19]
4	25-Epi-28-epi-cyasterone	Zhou et al. [18, 19]
5	24-Hydroxycyasterone	Zhou et al. [18, 19]
6	Sengosteron	Zhou et al. [18, 19]
7	Amaranth A	Zhou et al. [18, 19]
8	Precyaterone	Zhou et al. [18, 19]
9	Makisterone B	Zhou et al. [18, 19]
10	2,3-Isopropylidene cyasterone	Zhou et al. [18, 19]
11	2,3-Isopropylidene isocyasterone	Zhou et al. [18, 19]
12	Ecdysterone	Zhou et al. [21]
13	25R Inocosterone	Zhou et al. [21]
14	25S Inocosterone	Zhou et al. [21]

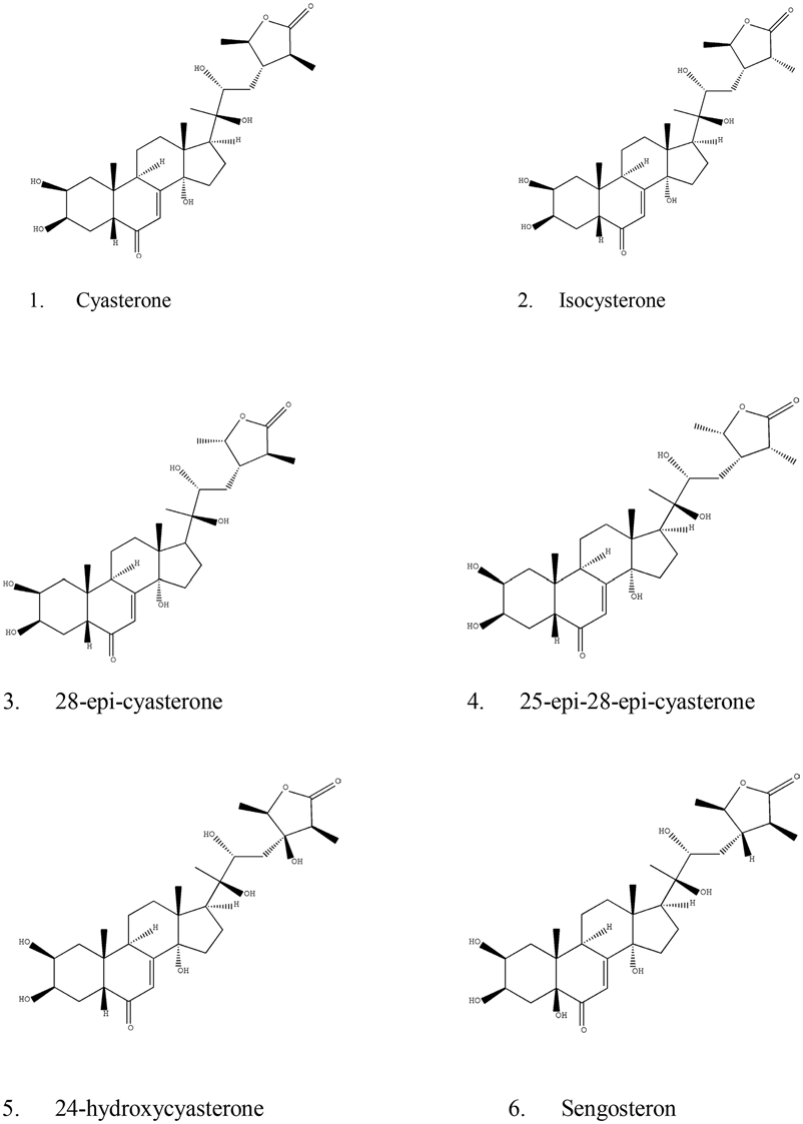
 
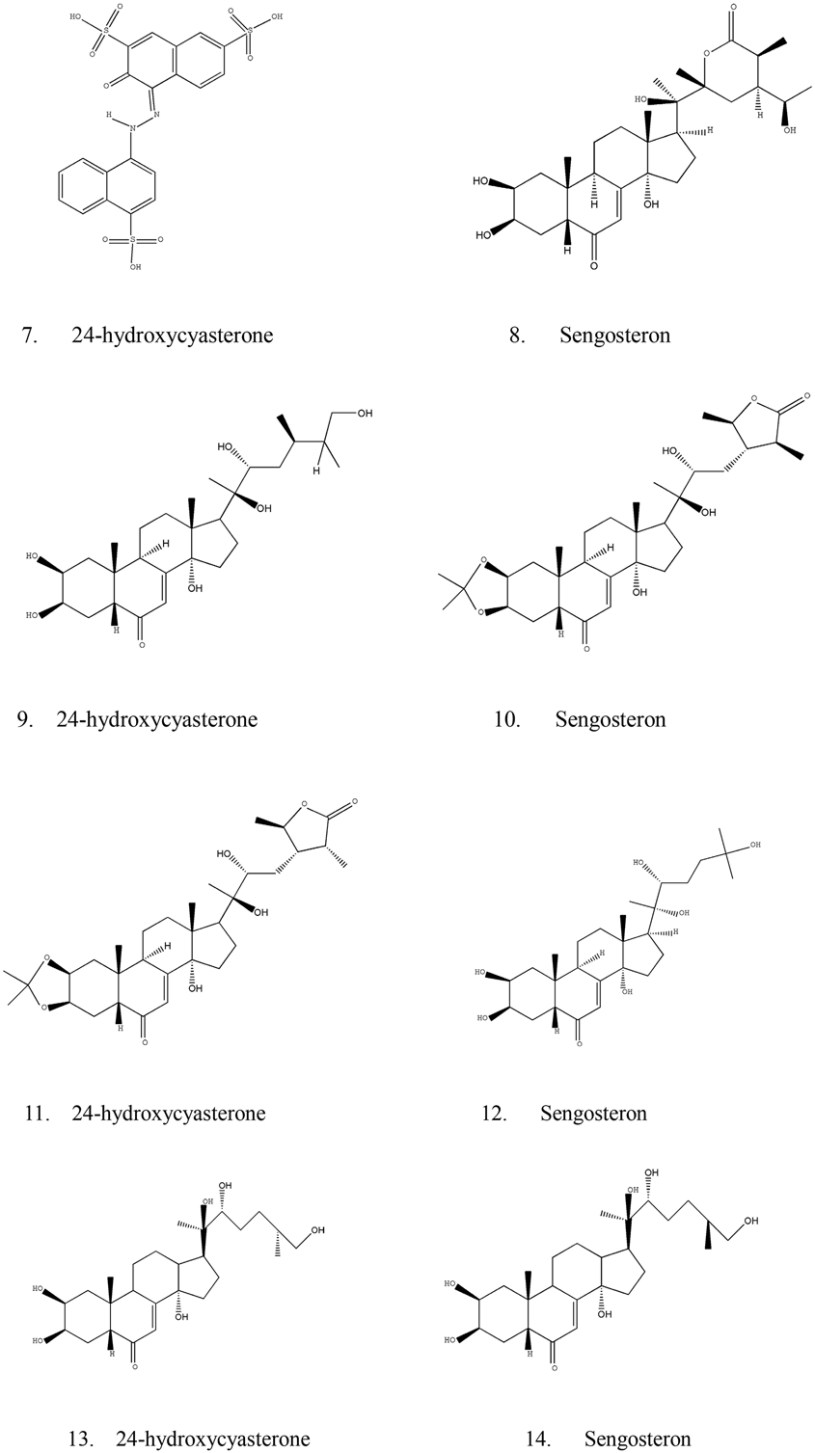

### Polysaccharide and other compounds

The polysaccharide of CR is a kind of bioactive polysaccharide extracted from the roots. It has a relative molecular weight of 1000–2200 and is a highly branched fructan. Modern pharmacological studies showed that *Achyranthes bidentata* polysaccharides can promote cellular immunity and antitumor properties and reduce peripheral leukocyte reduction induced by cyclophosphamide [22]. CR also contains glycine, valine, citramalic acid, ribonic acid, pipecolinic acid, *N*-carbamylglutamate, gluconic acid, 3-hydroxypropionic, glutaric acid, cellobiose, lactic acid, gluconic acid, xylitol, glutaric acid, pipecolinic acid, ribonic acid, mannose, oxalic acid, digalacturonic acid, lactic acid, 2-deoxyerythritol, acetol, 3-hydroxybutyric acid, *N*-carbamylglutamate, cellobiose, and palmitic acid [23, 24].

## Pharmacology

Cyathulae Radix possesses extensive pharmacological effects, including effects on the hematological system, urogenital system, and immune system; antifertility effects; anti-inflammatory effects; antitumor effects; senility-delaying effects; hypolipidemic effects; antihypertensive effects; antioxidation effects; and effects on climacteric syndrome, material metabolism, growth and development, and articulation [20]. In the following sections, the primary pharmacological abilities of CR are summarized and analyzed, as listed in Table 3.

**Table 3:** Preparation name, main compositions, traditional and clinical uses, and references

Preparation name	Main compositions	Traditional and clinical uses	References
Tianma Qufeng Bupian	Cyathulae Radix, Rhizoma Rehmanniae, Radix Angelica Sinensis, Notopterygii Rhizoma Et Radix, Angelica Pubescentis Radix, Radix Aconiti Lateralis Preparata, Cinnamomi Cortex, Gastrodiae Rhizoma, Eucommiae Cortex, Scrophulariae Radix, Poria	Warm kidney nourishing the liver, dispel wind and relieve pain	“Chinese Pharmacopoeia (2015)”, vol. 1^a^
Tianzhi Keli	Cyathulae Radix, Gastrodiae Rhizoma, Uncariae Ramulus Cum Uncis, Haliotidls Concha, Eucommiae Cortex, Taxilli Herba, Poria Cum Ligno Hospite, Polygoni Multiflori Caulis, Sophorae Flos, Gardeniae Fructus, Scutellariae Radix, Leonuri Herba	Calm the liver and suppress Yang, tonifying the liver and kidney, calm the nerves and improve intelligence	“Chinese Pharmacopoeia (2015)”, vol. 1^a^
Zhongfeng Huichun Wan	Cyathulae Radix, Prepared Angelicae Sinensis Radix, Prepared Chuanxiong Rhizoma, Carthami Flos, Persicae Semen, Salviae Miltiorrhizae Radix Et Rhizoma, Spatholobi Caulis, Lonicerae Japonicae Caulis, Trachelospermi Caulis Et Folium, Prepared Pheretima, Prepared Eupolyphaga Steleophaga, Lycopodium Clavatum Herba, Prepared Scolopendra, Prepared Leonuri Fructus, Scorpio, Prepared Clematidis Radix Et Rhizoma, Prepared Bombyx Batryticatus, Chaenomelis Fructus, Bungarus Parvus	Activating blood circulation to dissipate blood stasis, relaxing muscle and tendons and removing obstruction in the channels	“Chinese Pharmacopoeia (2015)”, vol. 1^a^
Mlaoji Wan	Cyathulae Radix, Auricularia Auricular, Angelicae Sinensis Radix, Paeoniae Radix Alba, Chuanxiong Rhizoma, Chaenomelis Fructus, Prepared Eucommiae Cortex, Dipsaci Radix, Atractylodis Rhizoma, Prepared Foeniculi Fructus, Aucklandiae Radix, Caryophylli Flos, Caryophylli Fructus, Prepared Olibanum, Poria, Smilacis Glabrae Rhizoma, Prepared Testudinis Carapax Et Plastrum	Tonifying the liver and kidney, dispelling dampness and dredging collaterals, activating blood and relieving pain	“Chinese Pharmacopoeia (2015)”, vol. 1^a^
Fufang Dianjixueteng Gao	Cyathulae Radix, Kadsurae Caulis Extract, Dipsaci Radix, Carthami Flos, Sojae Semen Nigrum	Activating blood and nourishing blood and the kidney	“Chinese Pharmacopoeia (2015)”, vol. 1^a^
Huoxue Zhuangjin Wan	Cyathulae Radix, Aconiti Radix Cocta, Carthami Flos, Draconis Sanguis, Olibanum, Myrrha, Eupolyphaga Steleophaga, Pheretima, Scorpio, Cinnamomi Ramulus, Ginseng Radix Et Rhizoma	Dispelling wind and activating blood, strengthen waist and tendons	“Chinese Pharmacopoeia (2015)”, vol. 1^a^
Chuanlong Guci Pian	Cyathulae Radix, Dioscoreae Nipponicae Rhizoma, Epimedii Folium, Cibotii Rhizoma, Rehmanniae Radix Praeparata, Lycii Fructus	Tonifying the kidney and strengthen the bones, activating the blood and relieve pain	“Chinese Pharmacopoeia (2015)”, vol. 1^a^
Tianjing Bushen Gao	Cyathulae Radix, Codonopsis Radix, Polygalae Radix, Epimedii Folium, Astragali Radix, Poria, Cibotii Rhizoma, Cistanches Herba, Rehmanniae Radix Praeparata, Angelicae Sinensis Radix, Morindae Offlcinalis Radix, Eucommiae Cortex, Lycii Fructus, Cynomorii Herba, Testudinis Carapacis Et Plastri Colla, Cervi Cornus Colla	Warming the kidney and enhance Yang, replenishing vital essence and blood	“Chinese Pharmacopoeia (2015)”, vol. 1^a^
Tongfengding Jiaonang	Cyathulae Radix, Gentianae Macrophyllae Radix, Phellodendri Chinensis Cortex, Corydalis Rhizoma, Paeoniae Radix Rubra, Alismatis Rhizoma, Plantaginis Semen, Smilacis Glabrae Rhizoma	Clearing heat and expelling damp, promoting blood circulation to remove meridian obstruction and relieving pain	“Chinese Pharmacopoeia (2015)”, vol. 1^a^
Xihong Tongluo Koufuye	Cyathulae Radix, Siegesbeckiae Herba, Carthami Flos	Dispelling wind and activating blood, dredging collaterals and relieving pain	“Chinese Pharmacopoeia (2015)”, vol. 1^a^
Wan Nian Chun Medicinal Liquor	Radix Cyathulae, Ginseng Radix et Rhizoma Rubra, Lycii Fructus, Illicii Cortex, Polygonati Odorati Rhizoma, Cynomorii Herba, Cynomorii Herba, Epimedii Folium, Salviae Miltiorrhizae Radix et Rhizoma, Carthami Flos, Prepared Cibotii Rhizoma, Prepared Atractylodis Macrocephalae Rhizoma	Invigorating qi and strengthening spleen, nourishing essence and nourishing kidney, expelling wind and activating blood circulation, strong muscles and bones	“Wei sheng bu yao pin biao zhun”, vol. 4^b^
Ren Shen Tian Ma Medicinal Liquor	Cyathulae Radix, Gastrodiae Rhizoma, Astragali Radix, Carthami Flos, Dioscoreae Nipponicae Rhizoma, Ginseng Radix et Rhizoma	Invigorating qi and activating blood circulation, relieving tendons and relieving pain	“Wei sheng bu yao pin biao zhun”, vol. 3^b^
Jian Nao Bu Shen Pills/Oral Solution	Cyathulae Radix, Ginseng Radix et Rhizoma, Cervi Cornu Pantotrichum, Penis Canitis, Cinnamomi Cortex, Polygalae Herba, Arctii Fructus, Rosae Laevigatae Fructus, Eucommiae Cortex, Lonicerae Japonicae Flos, Forsythiae Fructus, Cicadae Periostracum, Dioscoreae Rhizoma, Polygalae Radix, Ziziphi Spinosae Semen, Amomi Fructus, Angelicae Sinensis Radix, Ostreae Concha, Fossil Fragments, Poria, Atractylodis Macrocephalae Rhizoma, Cinnamomi Ramulus, Glycyrrhizae Radix et Rhizoma, Paeoniae Radix Alba, Amomi Fructus Rotundus	Strengthening the brain and nourishing the kidney, replenishing qi and strengthening spleen, tranquilizing and sedating the mind	“Wei sheng bu yao pin biao zhun”, vol. 21^b^
Qian Lie Shu Le Granules	Cyathulae Radix, Epimedii Folium, Astragali Radix, Typhae Pollen, Plantaginis Herba	Tonifying kidney and replenishing qi, removing stasis and promoting drenching	“Wei sheng bu yao pin biao zhun”, vol. 12^b^
Shen Gui Lu Rong Pills	Cyathulae Radix, Ginseng Radix et Rhizoma, Cervi Cornu Pantotrichum, Corni Fructus, Rehmanniae Radix, Rehmanniae Radix Praeparata, Paeoniae Radix Alba, Testudinis Carapax et Plastrum Praeparata, Trionycis Carapax, Asini Corii Colla, Eucommiae Cortex Praeparata, Dipsaci Radix, Asparagi Radix, Poria, Ziziphi Spinosae Semen, Succinum, Artemisiae Argyi Folium Praeparata, Citri Reticulatae Pericarpium, Alismatis Rhizoma, Myrrha Praeparata, Olibanum Praeparata, Corydalis Rhizoma, Carthami Flos, Croci Stigma, Achyranthis Bidentatae Radix, Celosiae Cristatae Flos, Halloysitum Rubrum, Cyperi Rhizoma, Glycyrrhizae Radix et Rhizoma	Tonifying qi and kidney, nourishing blood and regulating menstruation	“Wei sheng bu yao pin biao zhun”, vol. 3^b^
Shen Rong Gu Ben Huan Shao Pills	Cyathulae Radix, Ginseng Radix et Rhizoma, Aconiti Lateralis Radix Praeparata, Cinnamomi Cortex, Cuscutae Semen, Eucommiae Cortex, Curculiginis Rhizoma, Epimedii Folium, Cistanches Herba, Morindae Offlcinalis Radix Praeparata, Psoraleae Fructus Praeparata, Hippocampus, Achyranthis Bidentatae Radix, Actinolite, Actinolitum Brevifibrum, Astragali Radix Praeparata Cum Melle, Codonopsis Radix, Atractylodis Macrocephalae Rhizoma, Dioscoreae Rhizoma, Poria, Glycyrrhizae Radix et Rhizoma Praeparata Cum Melle, Rehmanniae Radix, Rehmanniae Radix Praeparata, Testudinis Carapax et Plastrum, Testudinis Carapacis et Plastri Colla, Asini Corii Colla, Polygoni Multiflori Radix Praeparata, Corni Fructus, Lycii Fructus, Ophiopogonis Radix	Nourishing kidney and Yang, replenishing qi and solid body, strong tendons and healthy bone, nourishing kidney essence to prevent seminal emission	“Wei sheng bu yao pin biao zhun”, vol. 11^b^
Zhuang Yao Xiao Tong Ye	Cyathulae Radix, Lycii Fructus, Epimedii Folium Praeparata, Morindae Offlcinalis Radix, Dioscoreae Nipponicae Rhizoma, Pheretima, Clematidis Radix et Rhizoma, Cibotii Rhizoma, Siegesbeckiae Herba, Mume Fructus, Cervi Cornus Colla, Pyrolae Herba, Chaenomelis Fructus, Myrrha, Syngnathus, Eucommiae Cortex	Strengthen waist and kidney, dispelling wind and dampness, dredging meridians and relieving pain	“Wei sheng bu yao pin biao zhun”, vol. 4^b^
Zhuang Gu Mu Gua Medicinal Liquor	Cyathulae Radix, Polygonati Odorati Rhizoma, Gardeniae Fructus, Chaenomelis Fructus, Angelicae Sinensis Radix, Notopterygii Rhizoma et Radix, Angelicae Pubescentis Radix, Citri Reticulatae Pericarpium, Periplocae Cortex, Chuanxiong Rhizoma, Gentianae Macrophyllae Radix, Carthami Flos, Taxilli Herba, Homalomenae Rhizoma, Leopard Bone	Dispelling wind, removing dampness, activating meridians and activating channels	“Wei sheng bu yao pin biao zhun”, vol. 20^b^
Tian Ma Zhui Feng Gao	Cyathulae Radix, Gastrodiae Rhizoma, Zaocys, Cinnamomi Ramulus, Pini Lignum Nodi, Mori Ramulus, Ephedrae Herba, Clematidis Radix et Rhizoma, Typhonii Rhizoma, Aconiti Radix, Aconiti Kusnezoffii Radix, Saposhnikoviae Radix, Dioscoreae Hypoglaucae Rhizoma, Menthae Haplocalycis Herba, Angelicae Pubescentis Radix, Angelicae Sinensis Radix, Uncariae Ramulus Cum Uncis, Schizonepetae Herba, Gentianae Macrophyllae Radix, Chuanxiong Rhizoma, Stephaniae Tetrandrae Radix, Zingiberis Rhizoma, Carthami Flos, Asari Radix et Rhizoma, Ligustici Rhizoma et Radix, Psoraleae Fructus, Notopterygii Rhizoma et Radix, Olibanum, Myrrha, Caryophylli Flos, Borneolum Syntheticum	Dispelling wind and removing dampness, activating blood circulation and dredging collaterals, dispersing cold and relieving pain	“Wei sheng bu yao pin biao zhun”, vol. 2^b^
Fu Ning Wan	Cyathulae Radix, Leonuri Herba, Codonopsis Radix, Rehmanniae Radix, Rehmanniae Radix Praeparata, Angelicae Sinensis Radix, Citri Reticulatae Pericarpium, Linderae Radix, Paeoniae Radix Alba, Paeoniae Radix Alba, Atractylodis Macrocephalae Rhizoma Praeparata, Cyperi Rhizoma Praeparata, Poria, Aucklandiae Radix, Perillae Folium, Asini Corii Colla, Amomi Fructus, Scutellariae Radix, Glycyrrhizae Radix et Rhizoma, Aquilariae Lignum Resinatum	Nourishing blood and regulating menstruation	“Wei sheng bu yao pin biao zhun”, vol. 1^b^
Han Shi Bi Tablets	Cyathulae Radix, Atractylodis Rhizoma, Lonicerae Japonicae Caulis, Pheretima, Forsythiae Fructus, Phellodendri Chinensis Cortex, Coicis Semen, Saposhnikoviae Radix, Dioscoreae Hypoglaucae Rhizoma, Mori Ramulus, Stephaniae Tetrandrae Radix, Clematidis Radix et Rhizoma	Dehumidification	“Wei sheng bu yao pin biao zhun”, vol. 16^b^
Qiang Li Tian Ma Du Zhong Capsules	Cyathulae Radix, Gastrodiae Rhizoma, Eucommiae Cortex, Aconiti Kusnezoffii Radix Cocta, Aconiti Lateralis Radix Praeparata, Angelicae Pubescentis Radix, Ligustici Rhizoma et Radix, Scrophulariae Radix, Angelicae Sinensis Radix, Rehmanniae Radix, Visci Herba, Notopterygii Rhizoma et Radix	Dispersing wind and activating blood circulation, relieving tendons and relieving pain	“Wei sheng bu yao pin biao zhun”, vol. 16^b^
Yu Xue Bi Granules	Cyathulae Radix, Clematidis Radix et Rhizoma, Carthami Flos, Salviae Miltiorrhizae Radix et Rhizoma, Olibanum, Myrrha, Chuanxiong Rhizoma, Angelicae Sinensis Radix, Curcumae Longae Rhizoma, Cyperi Rhizoma Praeparata, Astragali Radix Praeparata Cum Melle	Activating blood circulation to dissipate blood stasis, dredging collaterals and relieving pain	“Wei sheng bu yao pin biao zhun”, vol. 16^b^
Shi Re Bi Pian	Cyathulae Radix, Atractylodis Rhizoma, Lonicerae Japonicae Caulis, Pheretima, Forsythiae Fructus, Phellodendri Chinensis Cortex, Coicis Semen, Saposhnikoviae Radix, Dioscoreae Hypoglaucae Rhizoma, Mori Ramulus, Stephaniae Tetrandrae Radix, Clematidis Radix et Rhizoma	Dispelling wind and removing dampness, clearing away heat and swelling, dredging collaterals and relieving pain	“Wei sheng bu yao pin biao zhun”, vol. 21^b^
Hua Mo Yan Granules	Cyathulae Radix, Prunellae Spica, Ligustri Lucidi Fructus, Mahoniae Folium, Astragali Radix, Stephaniae Tetrandrae Radix, Coicis Semen, Smilacis Glabrae Rhizoma, Luffae Fructus Retinervus, Lycopi Herba, Salviae Miltiorrhizae Radix et Rhizoma, Angelicae Sinensis Radix, Siegesbeckiae Herba	Clearing away heat and removing dampness and activating blood circulation to dredge collaterals	“Wei sheng bu yao pin biao zhun”, vol. 18^b^
Xue Fu Sheng Tablets	Cyathulae Radix, Astragali Radix Praeparata Cum Melle, Angelicae Sinensis Radix, Paeoniae Radix Alba, Rehmanniae Radix Praeparata, Chuanxiong Rhizoma, Ligustri Lucidi Fructus, Ecliptae Herba, Poria, Dioscoreae Rhizoma, Trichosanthis Radix, Moutan Cortex, Alismatis Rhizoma, Glycyrrhizae Radix et Rhizoma, Rhei Radix et Rhizoma, Porcine Spleen	Invigorating qi and nourishing blood, nourishing yin and cooling blood, removing stasis and detoxifying	“Wei sheng bu yao pin biao zhun”, vol. 14^b^
Jin Mao Gou Ji Pills	Cyathulae Radix, Cibotii Rhizoma, Eucommiae Cortex, Chaenomelis Fructus, Mori Ramulus, Dipsaci Radix, Gentianae Macrophyllae Radix, Cinnamomi Ramulus, Pini Lignum Nodi, Piperis Kadsurae Caulis	Activating meridians, strengthening tendons and strengthening bones	“Wei sheng bu yao pin biao zhun”, vol. 8^b^
Dan Wang Granules	Cyathulae Radix, Salviae Miltiorrhizae Radix et Rhizoma, Vaccariae Semen, Smilacis Glabrae Rhizoma, Notoginseng Radix et Rhizoma, Scrophulariae Radix, Taraxaci Herba, Cinnamomi Ramulus, Astragali Radix, Myrrha, Alismatis Rhizoma, Spatholobi Caulis, Cirsii Japonici Herba, Gleditsiae Spina	Removing stasis and activating pulse, clearing away dampness and clearing heat, relieving swelling and relieving pain	“Xin Yao Zhuan Zheng Biao Zhun”, vol. 69^c^
Guan Xin Tai Pills	Cyathulae Radix, Ginseng Radix et Rhizoma, Astragali Radix, Rehmanniae Radix, Ophiopogonis Radix, Schisandrae Chinensis Fructus, Olibanum, Myrrha, Chuanxiong Rhizoma, Angelicae Sinensis Radix, Liquidambaris Fructus, Salviae Miltiorrhizae Radix et Rhizoma, Acori Tatarinowii Rhizoma, Leonuri Herba	Invigorating qi and nourishing heart and activating blood circulation	“Xin Yao Zhuan Zheng Biao Zhun”, vol. 32^c^
Zhuang Gu Zhi Tong Capsules	Psoraleae Fructus, Epimedii Folium Praeparata, Epimedii Folium Praeparata, Lycii Fructus, Ligustri Lucidi Fructus, Drynariae Rhizoma, Cibotii Rhizoma	Tonifying liver and kidney, strengthening bones and relieving pain	“Xin Yao Zhuan Zheng Biao Zhun”, vol. 83^c^
Qian Zhi Capsules	Cyathulae Radix, Notoginseng Radix et Rhizoma, Rubiae Radix et Rhizoma, Angelicae Dahuricae Radix	Activating blood circulation to stop bleeding, removing stasis and promoting growth, relieving swelling and relieving pain	“Xin Yao Zhuan Zheng Biao Zhun”, vol. 68^c^
Tong Luo Kai Bi Tablets	Cyathulae Radix, Strychni Semen Pulveratum, Angelicae Sinensis Radix, Carthami Flos, Chaenomelis Fructus, Schizonepetae Herba, Saposhnikoviae Radix, Scorpio	Dispelling wind and dredging collaterals and activating blood circulation to dissipate blood stasis	“Xin Yao Zhuan Zheng Biao Zhun”, vol. 33^c^
Ba Wei Tong Jing Tablets	Cyathulae Radix, Angelicae Sinensis Radix, Moutan Cortex, Paeoniae Radix Alba, Corydalis Rhizoma, Aucklandiae Radix, Persicae Semen, Cinnamomi Ramulus	Promoting blood circulation and regulating menstruation, removing blood stasis and relieving pain	“Guo Jia Zhong Cheng Yao Biao Zhun”, vol. of the surgery and gynecology^d^
Tong Feng Shu Capsules	Cyathulae Radix, Rhei Radix et Rhizoma, Plantaginis Semen, Alismatis Rhizoma, Stephaniae Tetrandrae Radix	Clearing away heat, dampness and detoxification	“Guo Jia Zhong Cheng Yao Biao Zhun”, vol. of the brain meridians and collaterals and limbs^d^
Gan Yue Pian Tablets	Cyathulae Radix, Codonopsis Radix, Polygoni Cuspidati Rhizoma et Radix, Salviae Miltiorrhizae Radix et Rhizoma, Bupleuri Radix, Hordei Fructus Germinatus	Soothing the liver and strengthening spleen, regulating qi and activating blood circulation	“Guo Jia Zhong Cheng Yao Biao Zhun”, vol. of the liver and gall^d^

^a^Cited from “Chinese Pharmacopoeia”

^b^Cited from “Wei sheng bu yao pin biao zhun”

^c^Cited from “Xin Yao Zhuan Zheng Biao Zhun”

^d^Cited from “Guo Jia Zhong Cheng Yao Biao Zhun”

### Effects on the hematological system

The characteristic pharmacological ability of CR on the hematological system has been researched on several levels, including the improvement of mesenteric microcirculation, enhancing the deformability of red blood cells (RBCs), reduction of platelet aggregation, decreasing the level of fibrinogen, promotion of wound healing, and lowering blood pressure and blood fat. From the point of view of TCM, CR has the function of activating blood and removing blood stasis. Cheng et al. [25] revealed that the water extracts of RC (WERC) at a dose of 5 g/kg could improve mesenteric microcirculation, decrease whole blood viscosity, and strengthen the deformation ability of RBC in acute blood stasis model rats. Wang et al. [26] reported that the addition of CR in Sijunzitang (Decoction of Four Mild Drugs, DFMD) accelerated the healing time and improved the healing rate for refractory wounds in a rat model of lower extremity. The mechanism of these effects is that DFMD increased the expression of vascular endothelial growth factor and the number of microvessels in the wound, which improved the anoxia and ischemia condition, accelerated the healing speed, and shortened the healing time of the wound [26]. After the addition of CR, the effects were stronger than those of DFMD alone. Further investigation revealed that DFMD and CR upregulated the synthesis and secretion of TGF-β_1_ and bFGF, accelerated fibroblast proliferation from the G0/G1 phase to the S phase, promoted the proliferation of fibroblasts and granulation tissues, and filled tissue defects [27]. Moreover, CR increased the proportion of fibroblasts in the S phase. Guo et al. suggested that CR has significant effects on hyperlipidemia and hepatic steatosis. WERC, components of 20% ethanol elution parts, and steroid components of CR could reduce the serum TC, TG, LDL-C, ALT, and AST values of hyperlipidemia rats; increase HDL-C value; reduce MDA levels in liver tissue; and increase SOD levels.

Moreover, Qi et al. [28, 29] revealed that the ethanol extracts of CR could reduce the blood pressure, decrease the activity of angiotensin-converting enzyme (ACE) and the apoptosis of cardiac cells, and block the effect of ACE in spontaneously hypertensive rats. In addition, a previous study [30] has also demonstrated that WERC could effectively alleviate left ventricular hypertrophy and improve myocardial remodeling in a dose-dependent manner.

### Effects on climacteric syndrome

Climacteric syndrome in women is due to the decline of ovarian function. Estrogen levels are significantly decreased by cardiovascular, nervous, and endocrine system disorders, leading to dysfunctional uterine bleeding, paroxysmal excess heat and sweating, vulvar and vaginal atrophy, osteoporosis, and other symptoms of the autonomic nervous system. Additionally, estrogen deficiency is the main cause of postmenopausal osteoporosis. Phytoestrogens, an alternative to estrogen, have attracted increasing attention in terms of their safety and efficacy [31].

Wang et al. [32] revealed that CR has a weak estrogen-like effect in ovariectomized rats. It could reduce the atrophy of the reproductive system induced by estrogen deficiency, improve lipid metabolism, and reduce body weight. It may be effective in treating climacteric syndrome. CR could increase uterine weight and improve endometrial atrophy in ovariectomized rats, indicating that it has the same function with endogenous estrogen. However, its stimulating effect on the endometrium is far less than that of estrogen. So the treatment of postmenopausal estrogen deficiency by using RC instead of estrogen can avoid the risk of inducing endometrial and breast cancer. Furthermore, CR shows a notable inhibitory effect on bone loss, improving bone biomechanical properties and preventing the occurrence of osteoporosis in ovariectomized rats.

In addition, CR reduces the degree of joint edema in rats with acute ventilated arthritis. The mechanism of the effect may be associated with downregulating the expression of NF-kB P65 and inhibiting the activity of NF-kB, thereby reducing inflammation [33].

### Effects on the immune system

Polysaccharides of CR (PCRs) possess adjuvant potential on cellular and humoral immune responses. Feng et al. found that PCRs (25, 50, 100 μg/mL) significantly enhanced the phagocytic capacity of peritoneal macrophages; splenocyte proliferation; and the activity of natural killer cells, cytotoxic T lymphocytes, and OVA-specific IgG, IgG1, IgG2a, and IgG2b antibody titers. Furthermore, PCRs promoted the levels of interleukin-2 (IL-2), interferon (IFN)-, and IL-4 in CD4(+) T cells and IFN- in CD8(+) T cells. In addition, PRCs enhanced the expression of CD40(+), CD80(+), and CD86(+) in dendritic cells (DCs). More importantly, PCRs downregulated the frequency of CD4(+)CD25(+)Foxp3(+)Treg cells. Taken together, these results suggested that PRCs could increase cellular and humoral immune responses by upregulating DC maturation and suppressing Treg frequency [34].

PCRs have attracted increasing attention from researchers. Li et al. [35] found that PCRs (2.5, 5, 10 g/kg) played a key role in enhancing the PFC response ability of mice immunized with SRBC, strengthening the cytotoxic activity of NK cells. However, the lymphocyte transformation rate did not change significantly. This finding showed that PRC could enhance the role of humoral immunity mediated by B cells, had no significant effect on T cell-mediated immunity, and could enhance the cytotoxic activity of NK cells. Wang et al. [36, 37] discussed the immune function of *Achyranthes bidentata* polysaccharides in vivo and in vitro. In vivo, PCR (25–100 mg/kg) could enhance delayed hypersensitivity and NK cell activity in normal mice and increase the carbon clearance rate in mice, number of antibody-producing cells, and macrophage phagocytosis of chicken RBCs with increasing polysaccharide concentrations. PCR could also significantly improve the number of white blood cells caused by cyclophosphamide (Cy) reduction. However, it had no effect on the transformation rate of spleen lymphocytes. In vitro, PCR in the concentration range of 10–300 μg/mL did not have a direct toxic effect on cells. It could promote the proliferation of B lymphocytes (P < 0.01) and enhance NK cell activity in mice peritoneal macrophages (P < 0.05) and neutral RBC activity (P < 0.01) with increasing polysaccharide concentration. However, it had no effect on the proliferation of T lymphocytes (P > 0.05). In the theory of TCM, CR possesses a tonic effect, which may be related to its polysaccharides.

### Antitumor effect

Song et al. [38] revealed that PCR has a certain inhibitory effect on mouse hepatoma cells. Ding [39] reported that PCR at doses of 1, 2.5, and 5 g/kg could effectively suppress mouse H22 hepatoma cells with inhibition rate of 21.99–42.21%. In S180 tumor-bearing mice, the inhibition rate was 10.00–48.08% with PCR dose of 1 and 5 g/kg. PCR not only had strong antitumor effects but also played a significant role in the recovery of peripheral leukocytes from mice (normal and tumor-bearing mice) induced by Cy. Zong et al. [40] revealed that 100, 200, and 400 μg/kg of PRC inhibited the growth of BEL-7402 cells and promoted cell apoptosis. The mechanism might be related to the upregulation of caspase-3 protein expression. Cao et al. [41] studied the effects of PCR on the tumor microenvironment of mice bearing HepG-2 cells. They administered 0.825 g/kg per day (low dose), 1.65 g/kg per day (middle dose), and 3.3 g/kg per day (high dose) of PCR in mice by gavage for 2 weeks. The results showed that PCR improved tumor immune inhibition, and high dose of PCR inhibited the expression of immunosuppressive factors in local tumor tissues and delayed tumor progression.

### Antioxidation and antiaging effects

Ding et al. [42] established a rat model of hepatocyte injury induced by H_2_O_2_ in vitro. They treated rats with 10 mg/mL of PCR with 73% purity. The results showed that PCR could reduce the increased levels of aminotransferase induced by H_2_O_2_ in rat stem cells, which might be related to the antioxidant effect of PCR. In a senescent mouse model, 0.25 mL of 55.6% pure *Achyranthes bidentata* polysaccharide was intragastrically administered, and 0.2 mL of d-galactose was injected subcutaneously [43]. The results showed that *A. bidentata* polysaccharides had stronger antioxidant activity than vitamin C, which is an excellent natural oxidant.

### Effects on material metabolism and growth

Cheng et al. [44, 45] revealed that CR contains molting sterones. Molting steroids increase glucose consumption within a certain concentration range, and the hypoglycemic effect of molting sterones decreases with increasing glucose concentration in the culture medium. The hypoglycemic effect of the molt is associated with hepatocytes and is non-insulin dependent. In a cell model of insulin resistance, ecdysterone can increase insulin sensitivity and improve glucose metabolism. As a reverse inhibitor, ecdysterone can improve insulin resistance caused by hyperglycemia, and its toxicity is very negligible.

### Effects on promoting angiogenesis

Cyathulae Radix is believed to stimulate menstruation and affect bone injury. Angiogenesis is actively involved in treating these illnesses. Zhou et al. reported that the whole extract of CR evidently increased cell migration. The open wound area was smaller than that of the control group by 14.9% after an 8 h treatment. The whole extracts of CR promoted angiogenesis mainly via stimulating the migration of endothelial cells in human umbilical vein endothelial cells in vitro. The authors found that several migration-related genes in zebrafish appeared to play pro-angiogenic roles of the CR extract in vivo. The results in human ECs are quite comparable to those in zebrafish. The mechanism of these effects is related to the enhanced gene expression of MMP9 and β-catenin by CR extract, revealing that matrix degradation and cell–cell adhesion are simultaneously triggered by the CR extract for stimulating angiogenesis. β-Catenin is a critical member of the canonical Wnt signaling pathway that is responsible for vascular sprouting, particularly for cell–cell adhesion. Moreover, it can trigger signal transduction for vascular remodeling and differentiation [46].

## Pharmacokinetics

To date, pharmacokinetic studies of CR extracts are few. Previous studies mainly focused on its ketosteroids such as cyasterone. In 2010, a study [47] reported that cyasterone is abundant in CR and can be used as a representative ingredient in pharmacokinetic studies. A rapid and sensitive method using reverse phase high-performance liquid chromatography (HPLC) was established to determine the cyasterone concentrations in rabbit plasma. The method provided bases for the study on CR pharmacokinetics. CR was traditionally considered as a guiding herb in TCM composite preparations and deeply valued by tens of millions of Chinese medicine practitioners. Tang et al. studied the effects of CR on paeoniflorin pharmacokinetics of Xuefu Zhuyu Tang in normal and blood-stasis model rats. After oral administration of solution with or without CR at a dose of 5 mL/kg, the peak plasma concentration (Cmax), peak time (Tmax), and half-life values of paeoniflorin were determined. The results indicated that the presence of CR in Xuefu Zhuyu Tang increased the Cmax of paeoniflorin from 0.099 to 0.363 mg/L and 0.228 to 0.315 mg/L in the normal and blood-stasis model group, respectively. Moreover, CR decreased the Tmax from 0.555 to 0.276 h in the normal group and the t1/2 from 0.853 to 0.501 h and 0.708 to 0.408 h in the two groups. Furthermore, the area under the concentration–time curve (AUC) was also determined. AUC0-∞ increased from 0.166 to 0.381 mg/L h in the normal group only. As a guiding herb, RC could significantly increase the absorption amount and rate of paeoniflorin in decoction and accelerated its elimination from the blood [48].

## Toxicity

Throughout its long history, CR has been generally considered to be a safe TCM in China [49]. No reports of poisoning have been reported in clinics. In a previous study, the toxicity of its ingredients such as ecdysone was found to be very low. The embryotoxicity of CR was also mentioned in the literature; however, a study on mice showed that its water decoction had no obvious induction effect of chromosomal aberration and embryonic micronucleus [50]. Recently, the acute toxicity of CR was reported in a dissertation [51]. The experimental results showed that after high-dose administration of water decoction, the mice exhibited neurotoxicity and gastrointestinal toxic reaction. Obvious toxic reactions were observed. Moreover, the maximum tolerance dose of samples from different origin ranged from 56.8 to 34.9 g/kg. This experimental result was different from the traditional cognition of safety of CR. However, the toxicity has not been clarified to date. Thus, further studies should be conducted to confirm the clinical innocuity of CR.

## Future perspectives and conclusions

CR has been used in China as an effective TCM for many years. Recently, it has been the subject of increasing interest. Many kinds of chemical constituents have been isolated and identified from this plant, and the pharmacological activity of its main chemical constituents such as alkaloids, steroids, and polysaccharides has been verified. Meanwhile, many traditional uses of this plant have been validated by pharmacological studies. In addition, previous animal investigations and in vitro studies have revealed that CR possesses significant effects on the hematological, urogenital, and immune systems. There is no doubt that continued progress has been made on various aspects of this plant in the past decade. However, further preliminary studies are needed to fully elucidate the mechanisms and constituents of this plant.

First, due to the complex introduction process, the mixture of CR and its adulterants has been widely used. This process has led to the common occurrence of adulterants in the herbal medicine market and misapplications in clinics, thus significantly affecting the value of CR. Therefore, cultivation management should be strengthened to remove the adulterants from the cultivation base. In addition, a unified identification is needed to control the variety and quality of this herbal medicine, and establishing a standardized finger print of this plant might be indispensable.

Second, the current pharmacological studies of this plant have primarily focused on several chemicals such as cyasterone and achyranthan. Evidence that can explain the special mechanism of action for the pharmacological activity of this plant is lacking. Thus, future studies should investigate more bioactive compounds and their mechanism of action and structure–function relationship. In addition, toxicity studies of aqueous extract are few, and no evaluations of target-organ toxicity have been documented. Furthermore, the experimental results about neurotoxicity and gastrointestinal toxicity contradict traditional understanding of CR. Therefore, further studies should devote more effort to investigate the compounds of CR and their toxicity.

Third, studies regarding the pharmacokinetics of CR are lacking. According to the only pharmacokinetic report of this plant, the marker compound cyasterone could be determined accurately in rabbit plasma via HPLC. Additionally, as a guiding-herb, CR could affect the pharmacokinetics of other medicines in Chinese compound formula. Therefore, future studies should investigate the pharmacokinetics of its main compounds.

Fourth, to date, studies on the parts of CR other than its roots are few. Other parts of this plant are worthy of multi-aspect research in the future. Thus, the chemical constituents and pharmacological effects of the aboveground parts should be investigated to reveal their possible medicinal potential.

Fifth, the traditional uses of CR include several processed products including the roots stir-fried with wine and salt. The traditional theory considered that the processing techniques with wine and salt could enhance its function of promoting menstruation and blood circulation and strengthening the muscles and bones [52]. Current investigations have found that the two processing techniques have significant effect on herbal chemical profiles, and several new compounds are generated in the process [53]. However, there is still not enough research to confirm the change of efficacy in the process. Moreover, special quality standards for processed CR are still lacking. Therefore, studies should investigate the chemical and pharmacological characteristics of processed CR systematically. Lastly, although CR is currently widely used and cultivated in many provinces of China, managing the variety of this plant and enhancing fundamental research are also important.

In addition, a similar Chinese medicine, Achyranthis Bidentatae Radix which is named Niuxi or Huainiuxi is used in the clinic. The original plants of Achyranthis Bidentatae Radix (Niuxi) and Cyathulae Radix (Chuanniuxi) looked similar and both of them are used to invigorate the circulation of blood. However, the main difference between these two herbs is efficacy. Achyranthis Bidentatae Radix is more suitable for tonifying the kidney and strengthen the muscles and bones, while Cyathulae Radix prefers promoting blood circulation and removing blood stasis, according to Chinese Pharmacopoeia 2015 Edition and teaching materials. Although this distinction is controversial now [54], it is used clinically according to this distinction.

Our present paper has systematically reviewed the traditional uses, origin and variety, phytochemistry, pharmacology, and toxicology of CR and provided comprehensive information on this plant. We hope that this review highlights the importance of CR and provides some directions for the future development of this plant.

## Abbreviations

CR: Cyathulae Radix
HPLC: high-performance liquid chromatography
