# Correction to: Review of traditional uses, botany, chemistry, pharmacology, pharmacokinetics, and toxicology of Radix Cyathulae

**DOI:** 10.1186/s13020-020-0294-1

**Published:** 2020-02-06

**Authors:** Yongliang Huang, Shanshan Wang, Li Liu, Wei Peng, Jiaolong Wang, Ying Song, Qianghua Yuan, Xing Yuan, Chunjie Wu

**Affiliations:** 1 Hospital of Chengdu University of Traditional Chinese Medicine, Chengdu, 610072 China; 2 Chengdu University of Traditional Chinese Medicine, Chengdu, 610075 China

## Correction to: Chin Med (2019) 14:17 10.1186/s13020-019-0237-x

In the original publication of this article [1], the English official name of the affiliation 1 has been changed to “Hospital of Chengdu University of Traditional Chinese Medicine, Chengdu, 610072, China”.
